# Point-of-Care Ultrasound for Physicians in Practice – A Training Model for Specialty Specific and Clinically Relevant Skill Development

**DOI:** 10.24908/pocus.v7i1.15433

**Published:** 2022-04-21

**Authors:** Lianne J Mclean, Resa E Lewiss

**Affiliations:** 1 Division of Emergency Medicine, Department of Paediatrics, The Hospital for Sick Children, University of Toronto Toronto Canada; 2 Emergency Medicine & Radiology, Thomas Jefferson University Philadelphia, PA USA

**Keywords:** physicians in practice, paediatric POCUS, international, in-person, asynchronous, online learning

## Letter

Point-of-care Ultrasound (POCUS) skills are required competencies for emergency medicine and paediatric emergency medicine training 1, 2, 3, 4. Over time, more specialties will require these skills of their graduates. Experienced physicians who completed their training before POCUS requirements may ask: How can I gain POCUS skills training and competency? In this perspective piece we describe in-person and asynchronous training programs available to these clinicians in practice. We highlight these programs due to their person-centred design: they maximise the needs of the learner, provide personalised education, and expose them to respected and established POCUS faculty and training centers.

POCUS learning revolves around 4 principles: image acquisition techniques, image interpretation, understanding of the clinical context, and integration into the patient care at the bedside. Some of these principles can be practised asynchronously, or exclusively online. Image interpretation programs such as ImageSim or Core Ultrasound providing large banks of practice images 5, 6. Short course workshops can create a foundation of acquisition skills. This is not enough as many physicians will need to supplement these with bedside skill development. Programs for physicians in practice allow training for physicians who lack local opportunities. While these programs vary in cost, scholarships and price reductions are possible and available depending on the practice location or specialty of the trainer. 

There are many free open access medical education (FOAMed) resources available; however, experienced clinicians are often seeking hands-on training, and deeper expertise to gain hospital privileges and competency. Short duration workshops are widely available in the form of pre-courses at conferences, or free-standing POCUS courses 7, 8. Some of these brief trainings even offer credentialing in core applications, however this may not translate directly to hospital privileges 7. It must be said that there is no evidence that these asynchronous and brief in person POCUS education offerings establish competency.

A longitudinal and in-person POCUS program has many advantages. Physicians in practice can gain confidence and experience in specific POCUS applications, can learn administrative skills on topics such as hardware, software, workflow, and quality improvement, and can gain a mentor and coach for hands-on POCUS skill competency. 

The Hospital for Sick Children in Toronto offers paediatricians the opportunity to train in person for 1-3 months. Trainers come from across Canada and around the world. The program’s relationship with its International Recruitment office allows provision of this program to learners globally. An existing paediatric emergency medicine POCUS framework serves as the core curriculum. The trainer is asked to provide specific objectives and goals. There is no clinical shift component or expectation. The focus is in developing POCUS skills and competency. As part of their time in the program, trainees have access to digital learning materials, to an asynchronous image interpretation program, and to participate fully in the training and educational schedule of the POCUS fellows and resident rotators. Focus is also placed on how to build local capacity at their home site, and in developing relationships across specialties or with hospitals in the trainer's region 9. 

The UC Irvine Sabbatical Program offers a one month program that is integrated into the existing POCUS curriculum. Final year medical students who have participated in a transformational ultrasound-prioritised medical education curriculum serve as the instructors for the physicians in practice 10, 11. Wilson et al published a sample four week POCUS curriculum mini-fellowship for physicians in practice 11. This was offered at the University of Colorado and now at Thomas Jefferson in Philadelphia 11. 

The Ultrasound Leadership Academy offers an asynchronous and remote learning option. This model allows clinicians to scan in their home environment. The 12-month remote learning curriculum includes one-on-one virtual support and quality assurance. There are in-person workshop opportunities. Models such as this offer training without requiring the participant to relocate 12. 

Physicians currently in practice can learn POCUS skills specific to their clinical needs. A longitudinal education program can support their needs in a more accessible and sustainable fashion. 

## Disclosures

REL serves on the medical advisory board of Echonous. REL is the director of POCUS at Thomas Jefferson University. LJM is the Director of POCUS at the Hospital for Sick Children. Both have taught and collaborated with directors and instructors of other programs mentioned.
